# The Itchy Truth About Scabies: A Case of Asymptomatic Carrier Transmission and Treatment Failure

**DOI:** 10.7759/cureus.50744

**Published:** 2023-12-18

**Authors:** Roksana Hesari, Nina Schur, Nicholas Tyndall, Thaddeus Chuchla, Nicky Gazy

**Affiliations:** 1 Osteopathic Medicine, Lake Erie College of Osteopathic Medicine, Bradenton, USA; 2 Dermatology, Western University of Health Sciences, Pomona, USA; 3 Dermatology, Beaumont Hospital, Trenton, USA

**Keywords:** skin disease/dermatology, recurrent rash, scabies, scabies treatment protocol, case report, itchy rash, sarcoptes scabiei var. hominis

## Abstract

Scabies, a common and highly contagious skin infestation, is caused by the mite* Sarcoptes scabiei *var.* hominis*. Identifying individuals with scabies often poses a diagnostic challenge, as its clinical features resemble other dermatologic conditions such as drug reactions, atopic dermatitis, and contact dermatitis. Furthermore, the cutaneous manifestations arise from delayed-type immunologic reactions to the mites and their byproducts, allowing some individuals to carry the mite without showing symptoms. The significant transmissibility of scabies, along with the potential for asymptomatic carriers, creates multiple treatment hurdles for cohabiting individuals, as the failure to treat all close contacts can result in re-infestation. This report presents the case of a 46-year-old Vietnamese male who suffered from a worsening erythematous, scaly, and pruritic rash for four months. Despite being prescribed topical corticosteroids by three different dermatologists, his rash persisted. Upon thorough evaluation, scabies was diagnosed. The patient was treated with scabicidal agents, which initially alleviated his symptoms; however, three weeks later, his symptoms resurfaced. Further investigation revealed that his wife was an asymptomatic carrier who had not received treatment. This case highlights the clinical features, pathogenesis, and treatment options for scabies while emphasizing the importance of promptly identifying and treating all close contacts.

## Introduction

Scabies is a highly contagious parasitic disease caused by the mite* Sarcoptes scabiei *var.* hominis*. In 2017, the World Health Organization declared scabies a neglected tropical skin disease and a serious health concern in many developing countries [1]. This particular parasitic disease affects approximately 150-200 million people each year, worldwide [2]. The most vulnerable populations at risk for scabies include young children, the elderly, immunocompromised individuals, and those who live in poor or overcrowded living conditions [3].

The scabies mite spreads through either direct skin-to-skin contact or contact with contaminated fomites. The adult female parasite, measuring 0.3-0.5 mm in length, burrows into the host's skin, creating 1-10 mm tunnels within the stratum corneum of its host, giving rise to the clinical signs and symptoms of the disease. Each day, approximately two to three eggs are laid by the mites, and on average, 10-15 adult female mites reside on the body of each infested individual [4]. For an individual to be infested through skin-to-skin contact, approximately five to 10 minutes of intensive contact must occur. Therefore, transmission through quick, casual modes of contact, such as hugs or handshakes, is unlikely. In the adult population, the most common mode of acquisition is through sexual contact. Notably, mites can survive outside the human body for 24-36 hours at 21°C and can live longer at higher humidity and lower temperatures [5]. Therefore, to ensure the elimination of these mites from contaminated fomites, washing temperatures exceeding 50°C is necessary [6].

The incubation period for scabies is approximately one month, during which an infested individual may not show any symptoms, but can unknowingly transmit mites to others [7]. Asymptomatic carriers are challenging to identify, necessitating a protocol requiring all cohabiting individuals, whether symptomatic or asymptomatic, to be treated for scabies to avoid re-infestation [3]. Here, an unusual case of scabies transmitted by an asymptomatic carrier is presented.

## Case presentation

**Figure 1:**
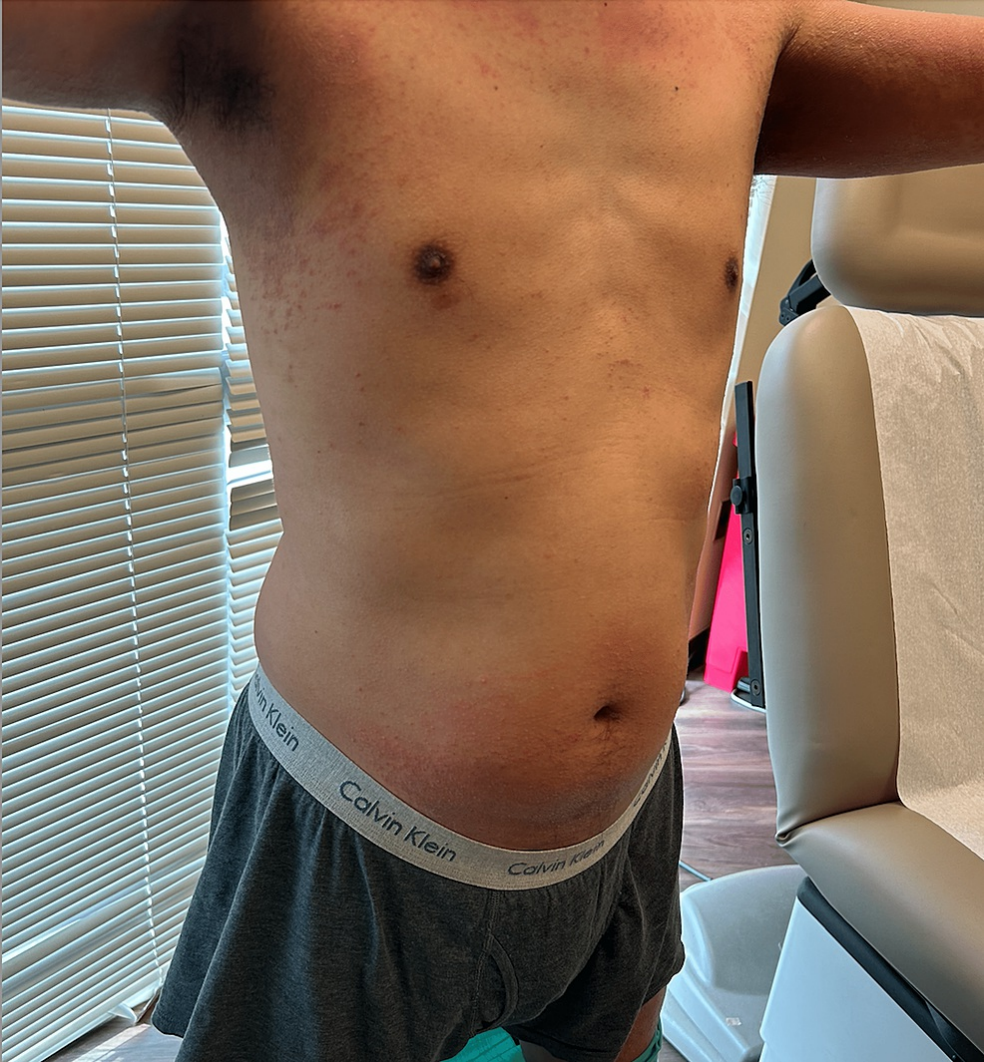
Scaly skin eruption with erythematous papules and patches on the waistband, neck, armpits, and upper lateral arms.

**Figure 2:**
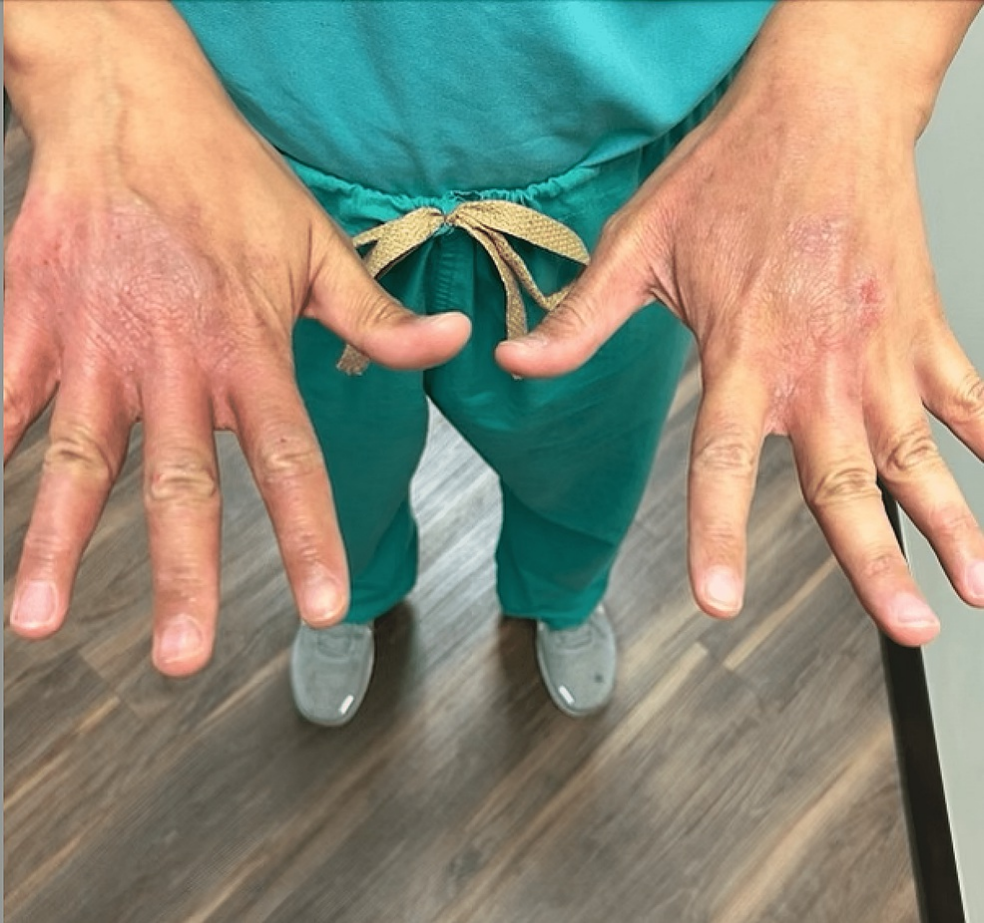
Excoriated skin with erythematous, scaly patches in the interdigital spaces, extending to the dorsum of the hands bilaterally.

A 46-year-old Vietnamese male podiatrist presented to an outpatient dermatology office for a persistent and worsening rash that had been troubling him for four months. The rash was characterized by erythematous, scaly papules and patches on the neck, bilateral upper extremities including the interdigital spaces, axillae, buttocks, navel, and scrotal area (Figure 1 and Figure 2). The patient reported that the rash was intensely pruritic, with a severity of 10/10, and worsened notably at night.

The patient had previously sought medical attention from three different dermatologists before this visit. Throughout the past four months, the patient attempted to treat his rash with topical corticosteroid creams that were prescribed for suspected eczema and contact dermatitis. However, these treatments did not resolve his rash. The patient had a history of internal cerebral artery calcifications and seizures, which required him to take levetiracetam 500 mg twice daily for the past eight years. Despite this long-term medication use, the patient did not report any adverse reactions to levetiracetam nor did he have any history of prior drug reactions. The differential diagnoses included scabies, atopic dermatitis, contact dermatitis, and symmetrical drug-related intertriginous and flexural exanthema (SDRIFE) secondary to levetiracetam.

Upon thorough evaluation and consideration of the differential diagnoses, the patient was diagnosed clinically with scabies infestation. The patient received an intramuscular injection of 40 mg of triamcinolone at the office to provide immediate relief from the severe itching and inflammation he was experiencing. The patient was also sent home with a prescription for permethrin 5% cream applied once a week for two weeks to target the scabies mites on the skin. He also received a single 15 mg oral tablet of ivermectin, and another dose was advised to be repeated two weeks later to further eliminate the mites. A one-pound jar of triamcinolone cream was prescribed for topical application for two weeks to address the inflammation and itching associated with the rash.

In light of the diagnosis, the patient's wife was also offered preemptive treatment to ensure containment within the household. Additionally, the patient was thoroughly instructed on the scabies treatment protocol, emphasizing the necessity of stringent hygiene measures, laundering clothing and linens in hot water, and vacuuming upholstery to minimize re-infestation. Following adherence to the treatment protocol, the patient observed a successful resolution of the rash within two weeks. Despite initially complying with the laundering of his sheets and rigorous hygiene measures, the patient continued to share a bed with his wife. Regrettably, his wife opted out of the preemptive treatment, citing her absence of symptoms as the reason for the refusal.

Five weeks after his initial visit, the patient returned with a relapse of his rash in the same areas. It was emphasized to the patient the importance of treating his wife as well as following proper scabies precautions to prevent re-infestation. Convinced by the advice, the patient persuaded his wife to undergo treatment for scabies. Both the patient and his wife were treated with permethrin and ivermectin. The treatment was administered as per the previous schedule.

Three weeks following the second round of treatment and compliance with scabies precautions, the patient's rash cleared up entirely, with no signs of recurrence. The follow-up examination revealed no scabies mites on the skin. Additionally, the erythematous and scaly pruritic patches were also resolved. Notably, the patient has not reported another recurrence of the rash since he initially presented to the clinic a year ago, indicating successful management of the scabies infestation.

## Discussion

The classic presentation of scabies is characterized by the appearance of serpiginous white lines and erythematous papules, accompanied by generalized pruritus that intensifies at night [3]. Typically, these distinctive features can be observed between the fingers and in the flexure of the wrist, elbows, axillae, umbilicus, beltline, buttocks, genitals, or breasts [8,9]. The diagnosis of a scabies infestation typically relies on recognizing the characteristic appearance and distribution of the rash. However, the gold standard of diagnosis is the visualization of mites, eggs, eggshell fragments, or mite fecal pellets by light microscopic examination after a skin scraping of the lesions. Dermoscopy is also utilized to search for patterns in the skin, including the "jet-with-contrail" sign, which represents the mite and its burrows, or the "delta-wing-jet" sign, which represents the head of the mite [2]. Currently, a test for scabies-specific molecular markers is not readily available for diagnosis, but its development could be helpful in cases of diagnostic uncertainty.

Certain patients, including those who are either debilitated, immunocompromised, or institutionalized, may present atypically when infested with scabies. According to a study on nursing home residents with confirmed scabies, a surprising 51% had not complained of itch, rash, or scratching. This indicated that more than half of the affected individuals did not show any overt signs of the infestation [10]. Another study described a cohort in which the majority of patients with scabies presented unusually with non-pruritic truncal papulosquamous dermatoses. The unique symptoms of these patients confounded the diagnosis and, in turn, delayed the treatment [11].

Two of the most frequently prescribed treatments for scabies include topical permethrin, a synthetic pyrethroid insecticide, and oral ivermectin, a macrocyclic lactone antibiotic. These two options have similar efficacy and are generally well-tolerated by patients. Permethrin 5% cream is considered the primary topical treatment in both the United Kingdom and the United States. It is effective against adult scabies mites (scabicidal) and their eggs (ovicidal), making it highly successful with just a single application. Nonetheless, it is common for the prescribed regimen to involve two applications in practice [12]. Ivermectin, on the other hand, has proven to be a successful oral treatment for scabies. Ivermectin is typically prescribed as a standard single dose of 200 μg/kg body weight. However, ivermectin does not possess ovicidal properties, meaning that a second dose may be necessary, ideally administered 14 days after the initial dose, to effectively eliminate newly hatched mites. When following the standard treatment of two doses, spaced two weeks apart, the cure rate is 97%, similar to the effectiveness of permethrin 5% cream [13]. In 2021, spinosad topical suspension 0.9% received approval from the United States Food and Drug Administration for treating scabies, marking one of the first new treatment options for this condition in the last three decades. Spinosad is believed to possess both scabicidal and ovicidal properties and is a promising novel treatment option for scabies in patients aged four and older [14].

There have been cases of pseudo-resistance to the treatment of scabies reported in the literature, which can be attributed to several factors. First, inadequate counseling of patients by medical professionals can result in patients not fully understanding the treatment protocol correctly [15]. Second, improper practices, such as insufficient quantity or duration of permethrin application, can result in the persistence of scabies infestations [16]. Third, solely treating a symptomatic patient is not enough to avoid scabies re-infestation. The effectiveness of treatment relies on addressing all close contacts of the patient at the same time, including family members and individuals sharing close living spaces. In the case of asymptomatic contacts, a single treatment with either permethrin or ivermectin is usually adequate [7]. Despite receiving treatment for scabies and being thoroughly counseled on the importance of adhering to the standard cohabitation treatment protocol, the patient in this report, unfortunately, experienced re-infestation. Non-compliance with the recommended guidelines led to the recurrence, underscoring the critical role of proper adherence to prevent such relapses.

Significantly, even when patients diligently adhere to all treatment instructions, they may still experience a complication known as post-scabies prurigo. This condition involves an eczematous delayed-type immunological reaction triggered by residual debris that remains in the skin after scabies treatment. The debris may consist of remnants from the mites or their fecal matter, contributing to the development of prurigo [16]. Post-scabetic prurigo is diagnosed based on several distinctive criteria. Firstly, there must be a documented history of successfully treated scabies. Secondly, the presence of red-brown, papulonodular, severely pruritic lesions must be observed in areas such as the groin (affecting 96.5% of patients) and axillae (37.9%) [17]. Thirdly, microscopic, histopathologic, and dermatoscopic examinations should show no evidence of mites, eggs, or scybala. Furthermore, scabicidal therapy proves ineffective in these patients, and the condition may persist for weeks or months. Post-scabetic prurigo tends to be more prevalent in male children and follows a chronic-relapsing course, with recovery often achieved through the use of potent topical corticosteroids [15,17].

Although post-scabetic prurigo is a relevant consideration in this context, it is essential to note that the patient described in the report faced a unique situation. This individual had been living with someone who did not undergo the recommended scabies treatment, necessitating the implementation of a repeated course of permethrin and ivermectin for both individuals. While these medications remain effective treatment options for scabies, instances of pseudo-resistance and post-scabetic prurigo emphasize the need for continued research and development of better treatment strategies.

## Conclusions

This case report reinforces the importance of early and accurate diagnosis, comprehensive patient education, and meticulous treatment adherence for the effective management of scabies. Continued efforts in public health initiatives and awareness campaigns are crucial, particularly in high-risk populations and resource-limited settings. By addressing challenges such as pseudo-resistance and post-scabetic prurigo, promoting preventive measures, and maintaining scabies in the differential for patients presenting with pruritus, we can advance towards a future where the burden of scabies is significantly reduced, ensuring better health and well-being for affected individuals worldwide. 
